# Stress-induced p53 drives BAG5 cochaperone expression to control α-synuclein aggregation in Parkinson's disease

**DOI:** 10.18632/aging.103998

**Published:** 2020-10-21

**Authors:** Huan-Yun Chen, Chin-Hsien Lin, Shu-Chun Teng

**Affiliations:** 1 Department of Microbiology, College of Medicine, National Taiwan University, Taipei, Taiwan; 2 Department of Neurology, National Taiwan University Hospital, Taipei, Taiwan; 3 Center of Precision Medicine, National Taiwan University, Taipei, Taiwan

**Keywords:** BAG5, cochaperone, p53, α-synuclein, Parkinson's disease

## Abstract

Parkinson’s disease (PD) is a common neurodegenerative disorder with the pathological hallmark of α-synuclein aggregation. Dysregulation of α-synuclein homeostasis caused by aging, genetic, and environmental factors underlies the pathogenesis of PD. While chaperones are essential for proteostasis, whether modulation of cochaperones may participate in PD formation has not been fully characterized. Here, we assessed the expression of several HSP70- and HSP90-related factors under various stresses and found that BAG5 expression is distinctively elevated in etoposide- or H_2_O_2_-treated SH-SY5Y cells. Stress-induced p53 binds to the BAG5 promoter directly to stimulate BAG5. Induced BAG5 binds α-synuclein and HSP70 in both cell cultures and brain lysates from PD patients. Overexpressed BAG5 may result in the loss of its ability to promote HSP70. Importantly, α-synuclein aggregation in SH-SY5Y cells requires BAG5. BAG5 expression is also detected in transgenic *SNCA* mutant mice and in PD patients. Together, our data reveal stress-induced p53-BAG5-HSP70 regulation that provides a potential therapeutic angle for PD.

## INTRODUCTION

PD is one of the most common neurodegenerative disorders in the aged society. The main pathological characteristics are caused by dysfunction of cellular proteostasis, leading to neuronal α-synuclein aggregation, thus progressively degenerating dopaminergic neurons. Increased levels of α-synuclein accumulate in Lewy bodies (LBs) and Lewy neurites (LNs), which correlate with the severity and progression of PD [1, 2]. Several lines of evidence have reported that the α-synuclein detrimental effects in neurons might be caused by cellular stress. Oxidative stress has been involved in inducing α-synuclein aggregation and plays an essential step in α-synuclein dimer formation [3, 4]. Moreover, severe hypoxia increases α-synuclein expression and oligomer formation [5]. Recently, α-synuclein was shown to link to genomic instability via ataxia telangiectasia mutated (ATM) and two of its downstream targets, histone H2AX and p53 [6–8]. Based on these findings, genomic instability caused by aberrant DNA damage responses, impaired DNA repair, reactive oxygen species (ROS) generation, and hypoxia have been hypothesized to constitute a set of pathological elements across PD [9]. Although many studies have revealed that α-synuclein expression is sensitive to external stimuli, the underlying mechanisms about how the cellular stresses contribute to the pathogenesis of PD are still elusive.

Misfolding, aggregation, and aberrant accumulation of proteins are hallmarks of many neurodegenerative disorders, including Parkinson's, Alzheimer's, prion, and Huntington's diseases [10, 11]. Protein aggregation causes cytotoxicity, neuronal injury, and cell death [12]. Chaperones and cochaperones execute protein folding and target aggregated proteins for refolding or degradation. The HSP70 and HSP90 chaperone and cochaperone systems play crucial roles in this process. Cochaperones such as J domain-containing HSP40 proteins recruit clients to HSP70 and stimulate ATP hydrolysis. Following the hydrolysis of ATP and protein folding, a nucleotide exchange factor then binds and opens the nucleotide-binding domain of HSP70 to allow ADP release that results in the release of the fully or partially folded substrate. The substrates then require HSP90 for complete folding or further refolding [13, 14].

A wide range of intrinsic or extrinsic stresses induces HSP70 and HSP90, but how cochaperones are regulated is not completely understood. We previously demonstrated that nutrient intake induces cochaperone Ids2 phosphorylation and HSP90 dysfunction [15]. Based on previous findings, we speculated that, under stress or in neurodegenerative disorders, HSP70- and HSP90-related factors may be stimulated to regulate proteostasis. In this study, we screened the expression of 21 HSP70- and HSP90-related factors under various stresses and found that only BAG5 expression levels are elevated in both etoposide- and H_2_O_2_-treated SH-SY5Y cells. BAG5, a nucleotide exchange factor of HSP70, was reported to be associated with enhanced protein aggregation in dopaminergic neurons [16–19]. BAG5 also displays an inhibitory function on HSP70 and Parkin, which may promote neurodegeneration [16, 20]. Here, we further identified that p53, a major stress-induced transcriptional factor, enhances BAG5 expression through direct binding to its promoter. p53 induces BAG5 expression in a dose-dependent manner. Overexpressed BAG5 binds HSP70 and reduces its refolding activity. Remarkably, α-synuclein aggregation in SH-SY5Y cells requires BAG5, and BAG5 expression is detected in a mouse PD model and postmortem brain tissues. Our findings provide further insight into the mechanisms of cellular stress-driven BAG5-mediated regulation in PD.

## RESULTS

### BAG5 expression is upregulated in etoposide- or H_2_O_2_-treated SH-SY5Y cells

**Figure 1:**
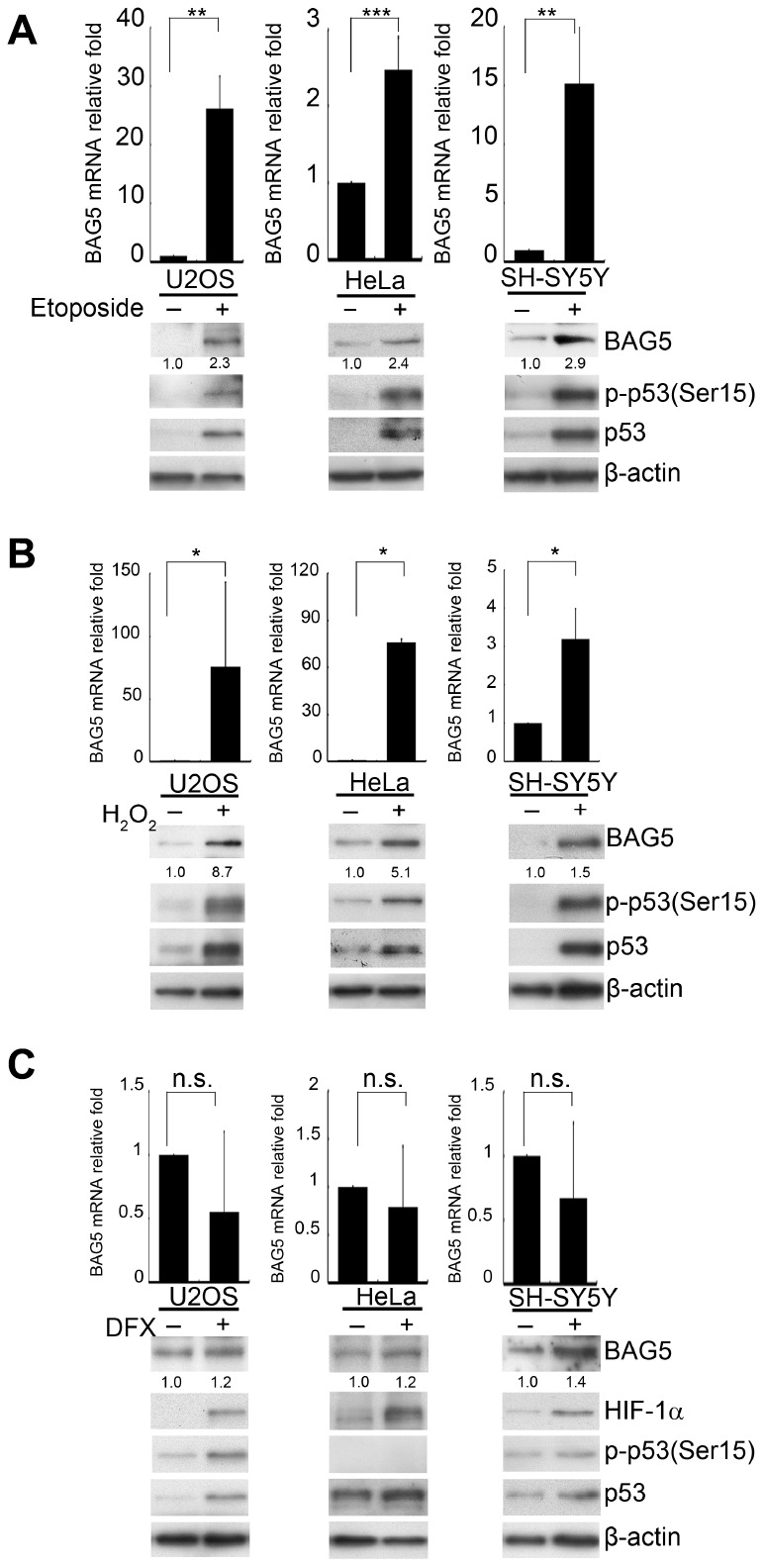
**BAG5 is upregulated in DNA damage- and oxidative stress-treated cells but not in hypoxia-treated cells.** (**A**–**C**) U2OS, HeLa, and SH-SY5Y cells were treated with 10 μM of etoposide for 48 h, 250 μM of H_2_O_2_ for 24 h, 10 μM of DFX for 48 h, or solvent. RNA was extracted, and BAG5 transcripts were detected by RT-Q-PCR. The relative fold in BAG5 mRNA expression was normalized to the GAPDH control and the solvent control (upper panel). The protein expression of BAG5, p53, HIF-1α, and β-actin was detected by Western blotting (lower panel). The relative fold in protein expression was normalized to that of the internal control β-actin and standardized with the solvent control. Error bars represent the SD of the means calculated using data from three independent experiments (Student’s t-test; *, *p* < 0.05, **, *p* < 0.01, ***, *p* < 0.001).

Stress responses are linked to the onset of many age-related frailties [21, 22]. Since genetic mutations can only explain the cause of 5~10% of PD, we speculated that stress-stimulated epigenetic transcriptional regulation may contribute to the formation of PD. Chaperones are highly expressed in response to stress. However, the roles of their related factors in neurodegenerative disorders are still not fully understood. Because α-synuclein aggregation is mainly resolved by the HSP70 system [23], we assessed the expression of the majority of the HSP70-related factors and some HSP90-related factors [24] under various stress conditions in neuronal and nonneuronal cells. We treated osteosarcoma U2OS, cervical carcinoma HeLa, and neuroblastoma SH-SY5Y cells with DNA damage (etoposide), oxidative stress (H_2_O_2_), and hypoxia-mimetic (deferoxamine, DFX) agents (Supplementary Figures 1–3). Interestingly, while different treatments induced distinct factors in different cells, only BAG5 mRNA levels increased in all three cells upon DNA damage or ROS treatment (Supplementary Figures 1, 2). Distinctive BAG5 upregulation was observed upon H_2_O_2_ treatment in SH-SY5Y cells (Supplementary Figure 2C). Examination of the transcripts of additional BAG members showed that only mRNA of p53-mediated BAG5 was consistently upregulated in etoposide- or H_2_O_2_-treated SH-SY5Y cells (Supplementary Figures 4, 5). To confirm whether BAG5 is indeed induced by stress stimuli, BAG5 mRNA and protein were further detected in drug-treated cells. A similar pattern was observed in the etoposide- and H_2_O_2_-treated cells but not in DFX-treated cells (Figure 1A–1C). p53 expression and phosphorylation, two indicators of the stress response, were also observed (Figure 1A–1C). Taken together, these data showed that etoposide and H_2_O_2_ upregulate BAG5 expression.

### Stress-driven p53 induces BAG5 activation

**Figure 2:**
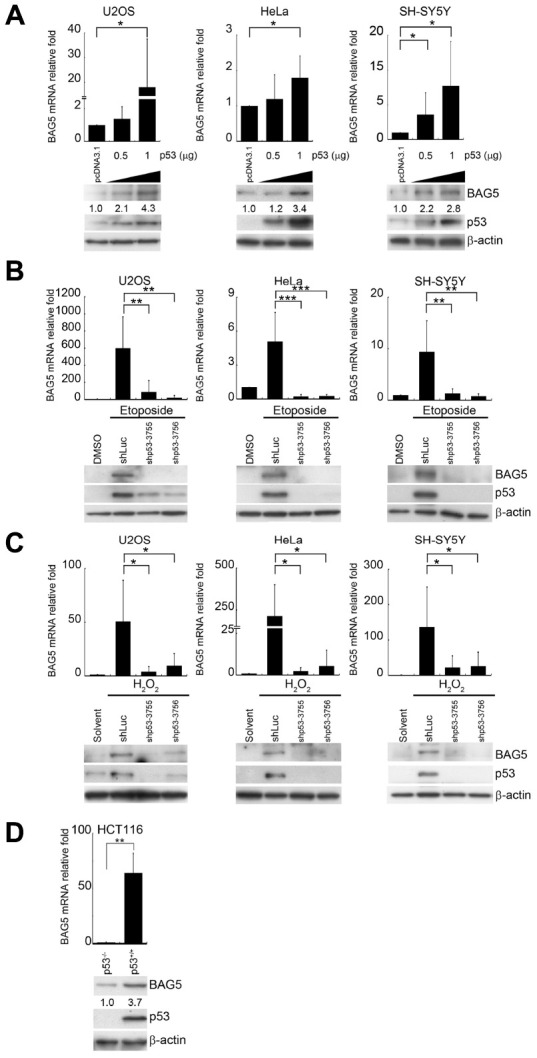
**p53 is responsible for BAG5 induction.** (**A**) U2OS, HeLa, and SH-SY5Y cells were transfected with various amounts of a p53-expressing plasmid (pcDNA3.1-p53). Expression levels of p53 and BAG5 were detected by Western blotting. β-Actin served as a loading control. After 24 or 48 h of pretreatment with etoposide (10 μM) or H_2_O_2_ (250 μM), p53 was repressed in etoposide-treated (**B**) and H_2_O_2_-treated (**C**) U2OS, HeLa, and SH-SY5Y cells by transfection of shp53-3755, shp53-3756, or shLuc control for 48 h and selecting with puromycin (2 μg/ml) for 48 h. The protein expression levels of BAG5, p53, and β-actin were detected by Western blotting. BAG5 transcripts were detected in shLuc and two p53 shRNAs knockdown cells. The expression levels of BAG5 transcripts were normalized to those of GAPDH and standardized with those of shLuc cells. (**D**) Total RNAs and cell lysates were harvested from HCT116 p53 wild-type (p53^*+/+*^) and null (p53^*-/-*^) isogenic colorectal cancer cell lines. BAG5 transcripts were detected by RT-Q-PCR. The relative fold in BAG5 mRNA expression was normalized to the GAPDH control and the vector control (upper panel). The protein expression levels of BAG5, p53, and β-actin were detected by Western blotting (lower panel). The relative fold in protein expression was normalized to the internal control β-actin and standardized with the vector control. Error bars represent the SD of the means calculated using data from three independent experiments (Student’s t-test; *, *p* < 0.05, **, *p* < 0.01, ***, *p* < 0.001).

p53 activation plays a central role in response to a range of cellular responses [25]. p53 expression and Ser15 phosphorylation under etoposide or H_2_O_2_ treatment (Figure 1A, 1B) suggest that p53 was transactivated under these stresses. To investigate whether p53 regulates BAG5 expression, we evaluated BAG5 expression levels in the presence of different amounts of p53 in U2OS, HeLa, and SH-SY5Y cells. Ectopic expression of p53 led to increases in BAG5 mRNA (Figure 2A, upper panel) and protein (Figure 2A, lower panel) in a dose-dependent manner. To test whether p53 is a crucial factor for BAG5 induction, knockdown of p53 by shRNAs was performed. While BAG5 expression was increased after etoposide or H_2_O_2_ treatment (Figure 2B, 2C and Supplementary Figures 4, 5), p53 knockdown caused a substantial decrease in BAG5 (Figure 2B, 2C and Supplementary Figures 4, 5). These data suggest that p53 is responsible for BAG5 induction. To further confirm the contribution of p53 to BAG5 activation, we tested BAG5 mRNA and protein expression levels in HCT116 wild-type (p53^*+/+*^) and p53 null (p53^*-/-*^) isogenic colorectal cancer cell lines. Both BAG5 mRNA and protein expression levels were detected in wild-type cells but were drastically repressed in p53 null cells (Figure 2D). These results strongly suggest that p53 is required for BAG5 induction.

### p53 binds directly to the BAG5 promoter

**Figure 3:**
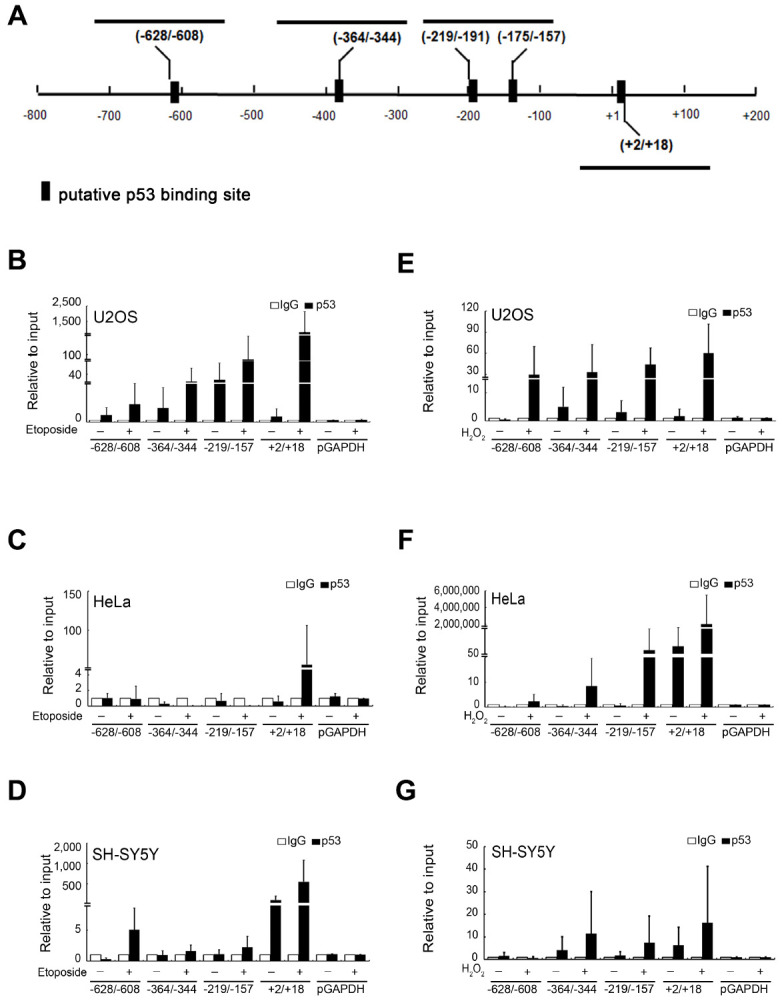
**Transcription factor p53 directly binds to the BAG5 promoter.** (**A**) A schematic diagram of human BAG5 promoter (positions −800 to +200 bps). The putative binding sites of the p53 transcription factor were predicted by ALGGEN-PROMO software. U2OS, HeLa and SH-SY5Y cells were treated with 10 μM of etoposide (**B**–**D**) for 48 h or 250 μM of H_2_O_2_ (**E**–**G**) for 24 h. Cell lysates were harvested post 24- or 48-h treatment, and DNA-protein complexes were immunoprecipitated using an anti-p53 antibody or the irrelevant rabbit IgG control. Q-PCR was performed for DNA-protein complexes to detect the DNA fragments at the BAG5 promoter (from -628 to -608, -364 to -344, -219 to -157, and +2 to +18). GAPDH promoter DNA (from -93 to +64) served as a negative control. The amount of immunoprecipitated DNA in each sample is represented as a signal relative to the total amount of input chromatin.

We further investigated the mechanism of p53-stimulated BAG5 expression. p53 is a major transcriptional factor in response to multiple stresses, and several posttranslational modifications of p53 activate its function [26, 27]. To explore whether p53 is directly involved in BAG5 transactivation, putative transcription-binding sites at the BAG5 promoter (NC_000014.9) were analyzed using the PROMO Transcription Factor Prediction System (http://alggen.lsi.upc.es/) (Figure 3A). Five potential p53 binding elements were observed at the BAG5 promoter between -800 and +200. To determine whether p53 binds directly to the *cis*-elements of the BAG5 promoter *in vivo*, chromatin immunoprecipitation (ChIP) was performed in U2OS, HeLa, and SH-SY5Y cells. As shown in Figure 3B–3G, etoposide and H_2_O_2_ induced p53 to bind to the BAG5 promoter. More importantly, p53 bound directly to the BAG5 promoter at +2 to +18 bp (Figure 3 and Supplementary Figure 6). Together, these results indicate that stress-induced BAG5 upregulation is mediated by p53 binding to the BAG5 promoter directly.

### Overexpressed BAG5 may result in the loss of its ability to promote HSP70

**Figure 4:**
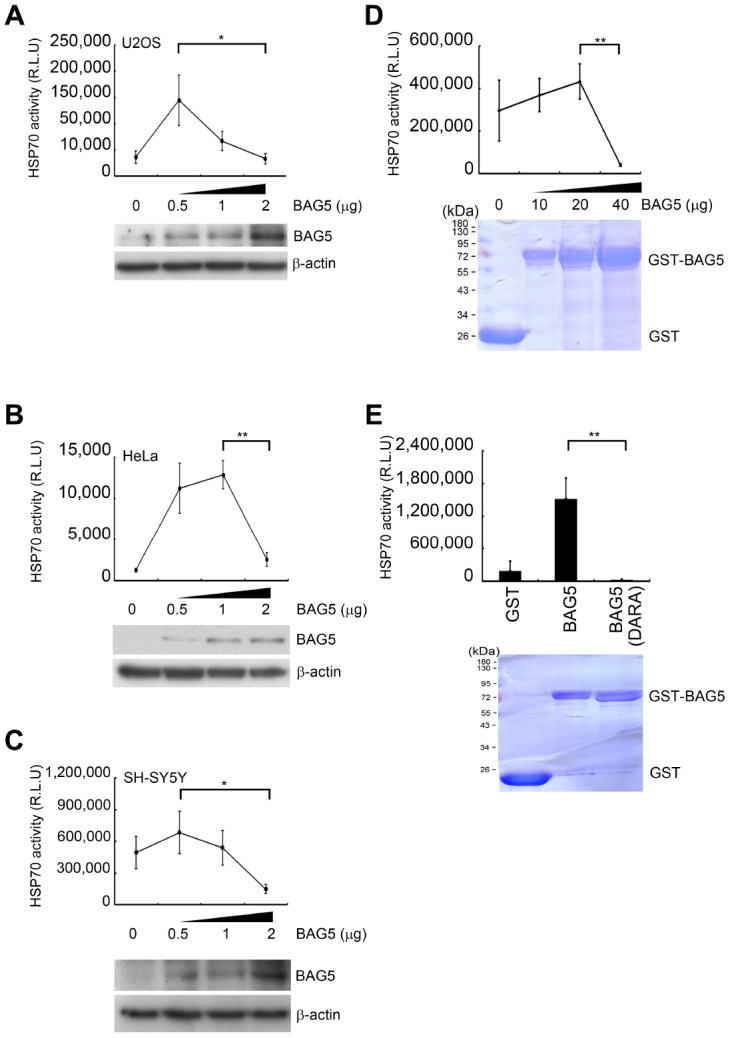
**Overexpressed BAG5 may result in loss of its function to promote HSP70 activity.** (**A**–**C**) U2OS, HeLa, and SH-SY5Y cells were transfected with various amounts of a BAG5-expressing plasmid (pCMV-Tag2B-BAG5). Expression levels of BAG5 were detected by Western blotting. β-Actin served as an internal control. (**D**–**E**) Recombinant GST fusion BAG5 and BAG5 (DARA) protein were purified and stained with Coomassie blue. The HSP70-mediated refolding activity was examined with denatured firefly luciferase in the presence of different concentrations of BAG5 (Student’s t-test; *, *p* < 0.05, **, *p* < 0.01).

HSP70, a chaperone regulated by BAG5, folds naïve proteins for their maturation. A previous study has shown that purified BAG5 can function as a nucleotide exchange factor of HSP70 for HSP70-mediated protein refolding [16, 28]. To determine whether BAG5 modulates HSP70 chaperone activity *in vivo*, we examined HSP70 activity under different levels of BAG5 expression. Using a Human HSP70/HSP40 Protein Refolding Kit [29] to investigate the effect of BAG5 on HSP70 in cells, we found that the refolding activity of HSP70 on heat-denatured firefly luciferase was enhanced in the presence of a lower level of BAG5 in all three cells (Figure 4A–4C). However, the high-level of BAG5 reduced the HSP70 refolding activity. These data indicate that overexpressed BAG5 may lead to loss of its promoting ability for HSP70 chaperone. To examine whether the BAG5-mediated biphasic phenotype was directly contributed by BAG5 itself or by its posttranslational modifications and/or associated factors, we further examined the effect of the *E. coli*-expressed recombinant GST-BAG5 on the HSP70-mediated refolding activity. The *in vitro* HSP70-mediated refolding activity was enhanced by a lower amount of GST-BAG5 but repressed by a high concentration of GST-BAG5 (Figure 4D). Conversely, GST-BAG5 (DARA) with defective binding to the HSP70 ATPase domain [16] failed to promote the refolding reaction (Figure 4E). To compare the relative levels of BAG5 between plasmid-mediated overexpression and stress-induced overexpression in SH-SY5Y cells, we quantified the intensity of Western blots [30]. A linear regression test using different amounts of the BAG5 plasmid in SH-SY5Y cells was performed. The amounts of etoposide- and H_2_O_2_-induced BAG5 calculated by the linear regression equation were 1.46 and 1.44 μg, respectively (Supplementary Figures 7), which are within the range displaying repression of HSP70 activity (Figure 4C). Taken together, these results imply that the overexpressed BAG5 may result in the loss of its ability to promote HSP70.

### Stress-induced BAG5 interacts with α-synuclein

**Figure 5:**
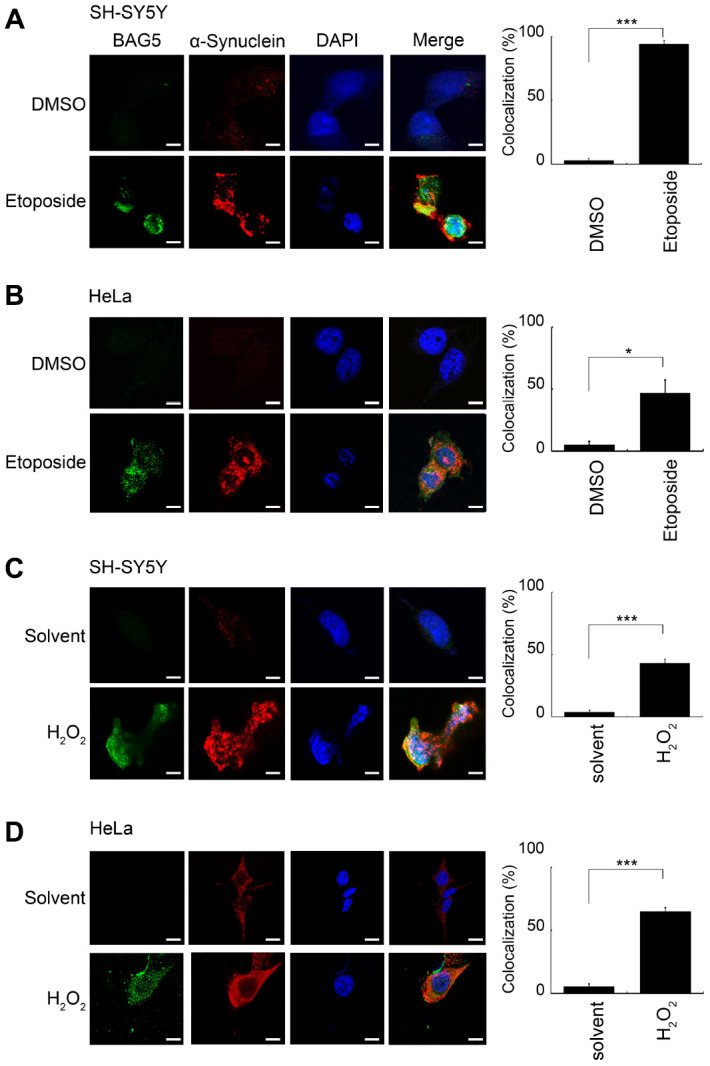
**BAG5 is activated upon stresses and colocalized with α-synuclein in the perinuclear compartment.** After 24 or 48 h of pretreatment with 10 μM of etoposide or 250 μM of H_2_O_2_, treated SH-SY5Y (**A**, **C**) or HeLa (**B**, **D**) cells were paraformaldehyde-fixed and stained using specific BAG5 (green) and α-synuclein (red) antibodies, and DAPI (blue) was used to stain the nuclear DNA. The images (1,260 x) were acquired using an LSM 510 Meta Confocal Microscope (Zeiss). The scale bar shows 20 μm. The percentage of cells with colocalization was determined by counting yellow blobs of at least 1,000 cells (Student’s t-test; *, *p* < 0.05, ***, *p* < 0.001).

Neuronal α-synuclein accumulation, which is a major component of LBs, is a central pathology of PD [1, 31]. Previous reports revealed that BAG5 expression is localized within dopaminergic neurons and LBs, which may colocalize with α-synuclein [16, 20]. To investigate the subcellular localization of stress-induced BAG5 and α-synuclein under cellular stresses, we visualized the intracellular distribution of the BAG5 and α-synuclein proteins in stress-induced HeLa and SH-SY5Y cells by confocal microscopy. As shown in Figure 5, compared to the solvent-treated control cells, BAG5 and α-synuclein were enriched in the majority of stress-exposed cells. Moreover, merged images indicated that BAG5 partially colocalizes with α-synuclein, especially at the perinuclear compartment, with some expression throughout the cytosol. Quantitative data showed that, after stress exposure, there are significant increases in the proportions of cells with BAG5 and α-synuclein colocalization (Figure 5, right panel).

To further examine the interaction between BAG5 and α-synuclein, we performed a Co-IP assay under stress treatments. U2OS, HeLa, and SH-SY5Y cells were treated with etoposide or H_2_O_2_, followed by Co-IP. BAG5 coimmunoprecipitated α-synuclein and HSP70 upon etoposide (Supplementary Figure 8A) and H_2_O_2_ (Supplementary Figure 8B) treatments. These results suggest that overexpressed BAG5 may be a partner of HSP70 and α-synuclein, which play roles in the response to stress.

### Stress-induced BAG5 expression results in α-synuclein aggregation

**Figure 6:**
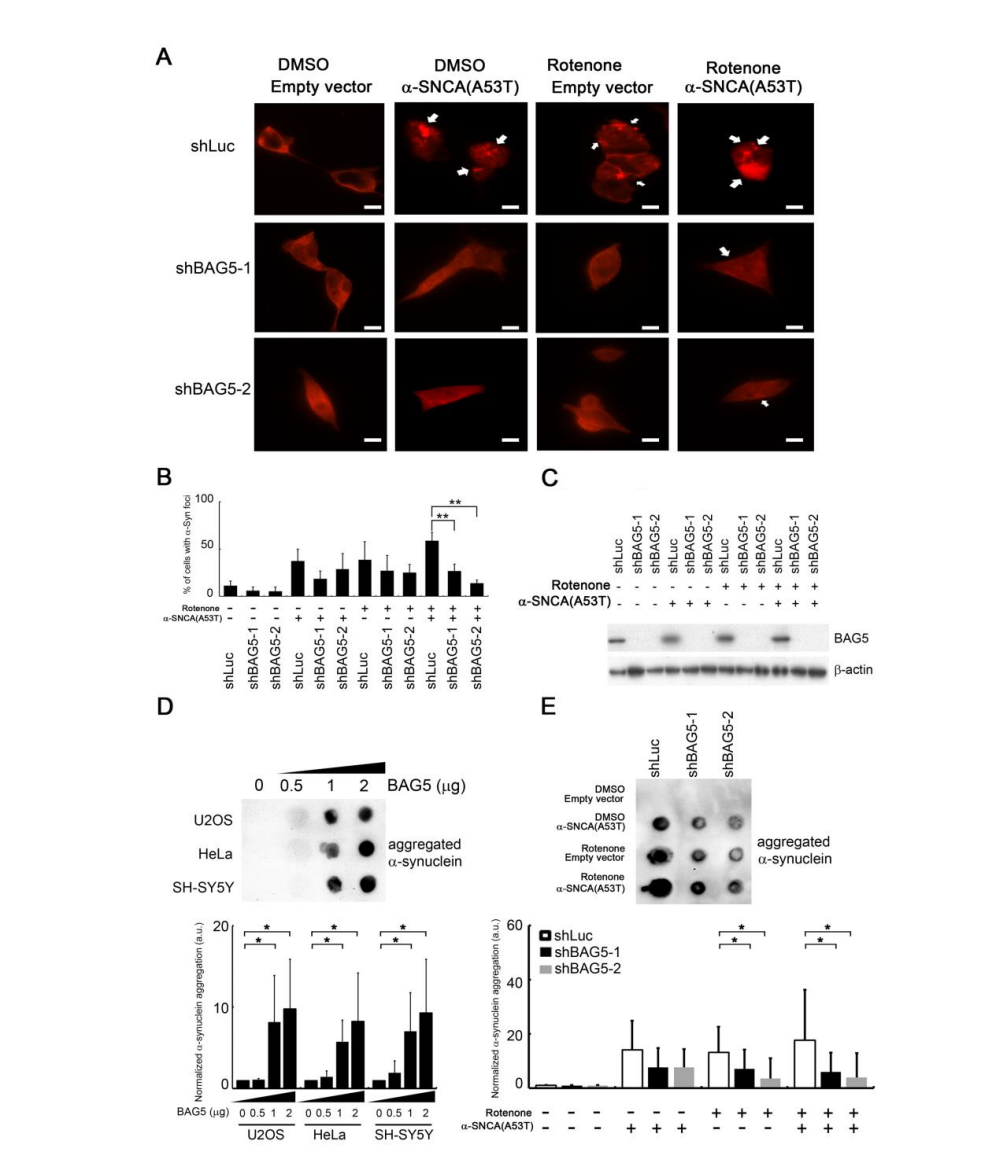
**BAG5 expression is crucial for α-synuclein aggregation.** (**A**) After rotenone treatment (10 μM) for 24 h, images of rotenone-treated α-synuclein (A53T)-expressing SH-SY5Y cells following BAG5 knockdown were captured. Arrows indicate α-synuclein-containing foci. Scale bar, 10 μm. (**B**) Quantified results in (**A**) are shown as the percentage of cells with foci (Student’s t-test; **, *p* < 0.01). (**C**) Knockdown of BAG5 in (**A**) was displayed by Western blot analysis. (**D**) BAG5 increases SDS-insoluble aggregation of α-synuclein in U2OS, HeLa, and SH-SY5Y cells. α-Synuclein aggregation was detected by the filter-trap assay in cells transfected with various amounts of a BAG5-expressing plasmid. The lysate was diluted in SDS and filtered through nitrocellulose membranes. α-Synuclein immunostaining was detected by the α-synuclein antibody. A representative image and the densitometry data are shown (a.u., arbitrary unit). The values of α-synuclein aggregation were normalized to the amount of aggregation in the empty vector control (Student’s t-test; *, *p* < 0.05). (**E**) SDS-insoluble α-synuclein aggregation in rotenone-treated and/or α-synuclein (A53T)-expressed SH-SY5Y cells is BAG5-dependent. α-Synuclein aggregation was detected by the filter-trap assay. The values of α-synuclein aggregation were normalized to the amount of aggregation in the vector or solvent control (Student’s t-test; *, *p* < 0.05).

Since overexpression of BAG5 suppresses HSP70 chaperone activity that may lead to α-synuclein accumulation, we further examined whether knockdown of BAG5 impairs α-synuclein accumulation and aggregation. To this aim, we transfected shRNAs against BAG5 in mutant α-synuclein (A53T)-expressed and/or rotenone-treated SH-SY5Y cells to trigger the formation of α-synuclein cytoplasmic foci [32–34], a phenomenon caused by α-synuclein aggregation. As expected, foci increased in mutant α-synuclein (A53T)-expressed and/or rotenone-treated cells (Figure 6A, arrow). Interestingly, α-synuclein foci were decreased following BAG5 knockdown in the rotenone-treated cells, suggesting that BAG5 expression is critical for α-synuclein aggregation (Figure 6A–6C).

In addition to microscopically visible aggregation, we further used a filter-trap assay to measure the amount of SDS-insoluble α-synuclein inclusions [33, 35]. High levels of BAG5 increased the amounts of SDS-insoluble aggregation of α-synuclein in all three cells (Figure 6D). Conversely, repression of BAG5 led to decreased SDS-insoluble α-synuclein aggregates in mutant α-synuclein (A53T)-expressed rotenone-treated SH-SY5Y cells (Figure 6E). Taken together, these results suggest that stress-induced overexpression of BAG5 increases the chance of α-synuclein aggregation and that loss of BAG5 expression is sufficient to mitigate the accumulation of insoluble α-synuclein aggregates.

### BAG5 expression and BAG5-α-synuclein association are increased in the brains of *SNCA* p.A53T mice and of human PD patient

**Figure 7:**
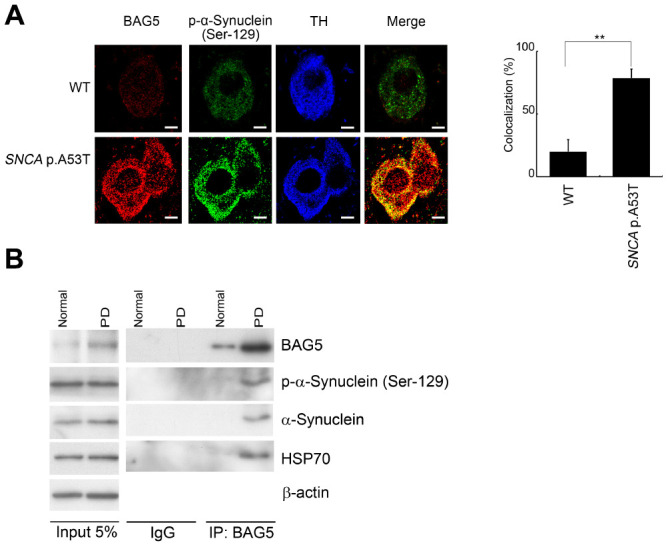
**BAG5 is increased and colocalized with α-synuclein in *SNCA* p.A53T mice and PD patients.** (**A**) Immunohistochemical staining of substantia nigra sections with a phospho-α-synuclein (Ser129) antibody revealed that the phospho-α-synuclein (Ser129) is expressed mainly in the substantia nigra. The expression of BAG5 (red) and p-α-synuclein (green) was detected mainly at the TH (blue)-positive excitatory synapse in *SNCA* p.A53T mice. The bars indicate 20 μm (Student’s t-test; **, *p* < 0.01). (**B**) Lysates were prepared from human normal control and PD brains. Immunoprecipitations were performed with an anti-BAG5 antibody. Immunoprecipitates were sequentially probed with phospho-α-synuclein (Ser129), anti-α-synuclein, and anti-HSP70 antibodies. Five percent of each lysate used for immunoprecipitation was loaded as input and probed with anti-BAG5, phospho-α-synuclein (Ser129), anti- α-synuclein, and anti-HSP70 antibodies. β-Actin was used as a loading control.

Because BAG5 expression was upregulated in stressed SH-SY5Y neuroblastoma cells, we next evaluated whether BAG5 expression is increased in a mouse model of PD [36]. We observed increased expression of BAG5 in tyrosine hydroxylase (TH)-positive nigral neurons of transgenic *SNCA* p.A53T PD mice, which express a p.A53T missense mutant form of the human α-synuclein (*SNCA*) gene under the control of the murine prion promoter (Figure 7A), compared to that in littermate wild-type mice. Increasing numbers of reports have demonstrated aberrant accumulation of phosphorylated α-synuclein at residue S129 in the brains of patients suffering from PD and of transgenic animal models of synucleinopathies [37–39]. Interestingly, BAG5 colocalized with phospho-Ser129 α-synuclein in the diseased mice (Figure 7A). These results provide a close link between BAG5 expression and α-synuclein aggregation. To further validate whether BAG5 interacts with α-synuclein along with PD progression, we performed Co-IP assays using human brain lysates from healthy controls and patients with PD. Consistently, we observed an increased expression level of BAG5 and more BAG5-associated HSP70 and α-synuclein in PD brain lysates compared to those in healthy control lysates (Figure 7B), indicating that more BAG5-HSP70- α-synuclein complex has formed in PD patients. These findings in human patients further imply that BAG5 interacts with α-synuclein and promotes α-synuclein accumulation along with PD progression.

## DISCUSSION

**Figure 8:**
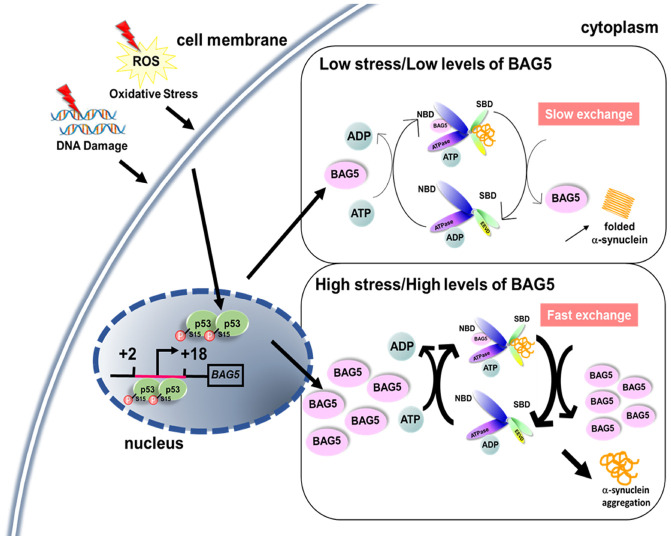
**A proposed role of stress- and p53-induced BAG5.** A schematic diagram shows that stress induces BAG5 expression through p53 binding at the BAG5 promoter. Under slight stress, a low level of BAG5 improves protein refolding efficiency by acting as a nucleotide exchange factor of HSP70. When the stress is too high, overexpressed BAG5 may accelerate the release of rashly folded clients from the substrate-binding domain (SBD) of HSP70, which causes unfolded protein aggregation.

BAG5, a member of the BAG family, is considered a pathogenic gene involved in dopaminergic neuronal degeneration [16, 20, 40, 41]. It was reported that physiological stress increases BAG5 expression [18, 42, 43]. However, our screen reveals the uniqueness of the BAG5 cochaperone in stress-mediated responses in SH-SY5Y cells. The BAG5-HSP70 interaction reduces cellular HSP70-mediated folding activity and increases α-synuclein aggregation (Figure 8). These *in vitro* findings were further supported by the colocalization of BAG5 and phospho-Ser129 α-synuclein in (TH)-positive nigral neurons in PD mice and by the BAG5-α-synuclein interaction in PD patients’ brain lysates. The findings support evidence of BAG5 dependency in α-synuclein dyshomeostasis, suggesting a role for BAG5 in the pathogenesis of PD.

p53 is characterized as a transcription activator of several cellular target genes in DNA repair [44], cell cycle arrest [45], senescence [46], and apoptosis [25] pathways. In our study, p53 acts as an activator of a cochaperone through direct binding to its promoter. These results offer an insight into the mechanism of BAG5 induction by stress to modulate chaperone activity for protein folding. As a nucleotide exchange factor, BAG5 can provide cellular protection from stresses by promoting protein folding [28]. Recent studies have also demonstrated the relationship between BAG5 and PD-related proteins, including PINK1 and DJ-1, further strengthening the role of BAG5 in dopaminergic neuron protection [17, 19]. Our Co-IP and immunofluorescence experiments demonstrate that BAG5 interacts with α-synuclein. However, a high concentration of BAG5 reverses its function in HSP70. Therefore, BAG5 regulates HSP70 activity in a biphasic manner. The balance between BAG5 dosage and HSP70 activity is critical during protein folding. Under slight stress, a low level of BAG5 may stimulate HSP70 activity by acting as an important stress-induced backup nucleotide exchange factor of HSP70 [28]. However, when the stress is too high for cells to tolerate, BAG5 represses cellular HSP70-mediated folding activity (Figure 8). In agreement with our data, an elevated concentration of the other nucleotide exchange factor, HSP110 (HSP105 in human and Apg2 in mouse), also reduces HSP70 activity probably by the premature release of incompletely folded substrates [47–50]. Alternatively, a high concentration of nucleotide exchange factors might reinforce the binding between nucleotide exchange factor and HSP70, which might generate a steric clash for the HSP70-J domain protein interaction and further prevent the optimal regulation in the next round of client recruitment [51]. Hence, balanced BAG5 expression is crucial for proper protein folding in stressed neuronal cells.

Previous studies demonstrated that purified recombinant BAG5 promotes HSP70 refoldase activity *in vitro* [28], while cellular BAG5 inhibits HSP70-mediated prevention of Parkin aggregation in HEK293T cells [16]. This seeming discrepancy points to the possibility that either additional cellular factors may be required for BAG5-mediated protein folding or the experimental conditions of the *in vitro* and *in vivo* chaperone assays employed may not be comparable. Here, we conducted the HSP70 refoldase assay using a range of concentrations of BAG5 purified from *E. coli* or of lysates with BAG5 overexpression, which allowed us to document BAG5-mediated HSP70 regulation. The biphasic HSP70 chaperone activity regulated by the recombinant BAG5 may rule out the possibility of additional cellular factors or posttranslational modifications, but the results indicate that the concentration of BAG5 itself determines its function *in vivo*.

A hallmark of PD is the widespread pathological accumulation of misfolded α-synuclein clumps in nerve cells [1]. Clearance of the abnormal aggregation requires proper degradation mechanisms, including chaperone-mediated autophagy, macroautophagy, and ubiquitin-proteasome pathways [52]. However, cellular factors involved in α-synuclein aggregation and clearance are still elusive. Here, we revealed the significant role of BAG5 in α-synuclein aggregation. A previous report found that BAG5 can inhibit CHIP-mediated α-synuclein ubiquitination to mitigate α-synuclein clearance [40]. In addition to ubiquitin-mediated regulation, we discovered another type of regulation to inhibit HSP70 activity that further induces α-synuclein aggregation. Therefore, BAG5 may utilize multiple pathways to induce α-synuclein clumps.

Interestingly, there are growing pieces of evidence that show that BAG5 promiscuously targets a range of PD-related proteins. BAG5 interacts with DJ-1 and thus inhibits DJ-1-mediated protective activity [19]. BAG5 directly binds PINK1 and regulates PINK1 degradation by inhibiting its ubiquitination [17]. BAG5 has an inhibitory effect on Parkin [16, 20]. Recently, BAG5 has also been found to regulate the bi-modal activity of Parkin, promoting cell death by suppressing Parkin-dependent mitophagy and enhancing Parkin-mediated Mcl-1 degradation [53]. Moreover, according to the protein-protein interaction arrays, BAG5, Rab7L1 (RAB7, member RAS oncogene family-like 1), and cyclin G-associated kinase may form a complex with the PD-related protein LRRK2 [41]. Based on all these findings, BAG5 seems to prefer to interact with PD-associated proteins to modulate their homeostasis. We speculate that the BAG5-mediated folding pathway may control the stabilities of other proteins.

In conclusion, we demonstrate that stress-driven BAG5 interacts with HSP70 and negatively regulates the activity of HSP70, attenuates the chaperone function, and promotes α-synuclein aggregation. Thus, it is conceivable that BAG5, by affecting HSP70 activity, significantly contributes to PD pathogenesis. These findings provide important implications for understanding the pathogenesis of PD and future novel therapeutics.

## MATERIALS AND METHODS

### Cell culture and reagents

U2OS, HeLa, and HCT116 p53 wild-type (p53^*+/+*^) and null (p53^*-/-*^) cell lines were maintained in DMEM containing 10% FBS and antibiotics (100 U/mL penicillin, and 100 μg/mL streptomycin) at 37°C with 5% CO_2_. Human neuroblastoma SH-SY5Y cells were cultured in DMEM/F12 (44.5/44.5%) supplemented with 10% FBS and antibiotics (100 U/mL penicillin, and 100 μg/mL streptomycin) at 37°C with 5% CO_2_. Cells were treated for the indicated time points with the final concentration of 10 μM/ml DNA-damaging drug etoposide (Sigma-Aldrich, USA), 250 μM ROS inducer H_2_O_2_ (Kanto Chemical Co., Japan), 10 μM hypoxia-mimetic DFX (Sigma-Aldrich, USA) and 10 μM electron transport chain inhibitor rotenone (Sigma-Aldrich, USA).

### Preparation of RNA, reverse transcription, and quantitative polymerase chain reaction (Q-PCR)

RNAs were extracted from cellular and plasma samples using TRIzol reagent (Invitrogen, USA). RNA reverse transcription was carried out using a Maxima First Strand cDNA Synthesis Kit (Thermo Scientific, USA) according to the manufacturer’s protocol. Real-time PCR was performed using the 2X SYBR Green PCR Master Mix (Kapa Biosystems, USA) on a CFX Manager system (Bio-Rad, USA). Q-PCR was performed in duplicate in each experiment following the standard protocol. Primer sequences for transcripts are shown in Supplementary Table 1.

### Western blotting and antibodies

Cells were harvested in cell lysis buffer, and proteins were separated by 10% SDS-PAGE. The target proteins were detected using enhanced chemiluminescent reagent (GE healthcare, USA). The antibodies used for immunoblotting were anti-BAG5 (Santa Cruz, USA), anti-p53 (Santa Cruz, USA), anti-HIF-1α (BD Biosciences, USA), anti-α-synuclein (Genetex, USA), anti-HSP70 (Santa Cruz, USA), anti-phospho-p53 (Ser15) (Cell Signaling, USA), anti-phospho-α-synuclein (Ser129) (Abcam, UK), and anti-β-actin (Sigma-Aldrich, USA). The autoradiographic films of Western blots were scanned by a Microtek ScanMarker i900, and the protein bands were quantified by ImageJ software. To convert the amounts of BAG5 from etoposide and H_2_O_2_ stimulation to the equivalent BAG5 expression from plasmid transfection, Western blots were quantified as above. Linear regression was calculated using the ImageJ scores from different amounts of BAG5 plasmid transfection after normalization with the β-actin scores. The amounts of etoposide- and H_2_O_2_-induced BAG5 were then calculated by the linear regression equation [30].

### Plasmids, cell transfection, and RNA interference

The DNA region encoding human p53 was cloned into the pcDNA3.1-Myc vector. The cDNA of α-synuclein was amplified from SH-SY5Y cDNA and cloned into the pGEM-T Easy vector (Promega, USA). The α-synuclein A53T mutation was introduced by PCR using a Phusion™ High-Fidelity Kit (Thermo Scientific, USA). The full-length human A53T α-synuclein cDNAs were further cloned into the pcDNA3.1-Myc vector. The full-length BAG5 fragment was cloned into the pCMV-Tag2B vector at the EcoRI/HindIII sites to create an N-terminal flag tag. The full-length human BAG5 or BAG5 (DARA) was cloned into the pGEX-4T-1 vector [15]. Cells were transfected with T-Pro Non-liposome Transfection Reagent (T-Pro Biotechnology, Taiwan) according to the manufacturer’s instructions. Plasmids pLKO.1- and pLKO_TRC005-based short hairpin RNAs were prepared by the National RNAi Core Facility in Taiwan. For shRNA-mediated knockdown of p53 or BAG5, cells were transfected with the shp53 or control shLuc shRNAs using T-Pro NTR II Reagent (T-Pro Biotechnology, Taiwan) for 2 days and selected in 2 μg/ml puromycin for 24 or 48 h. The shRNA targeting sequences are shown in Supplementary Table 1.

### Chromatin immunoprecipitation (ChIP) assays

ChIP assays were performed as previously described [54]. Briefly, etoposide- or H_2_O_2_-treated U2OS, HeLa, and SH-SY5Y cells were harvested and cross-linked with 1% formaldehyde. The DNA-protein complex was precipitated by the p53 antibody (Santa Cruz, USA). The primers used to detect immunoprecipitated DNA are shown in Supplementary Table 1.

### Immunofluorescence and confocal microscopy

Stress-induced cells were seeded onto glass coverslips (Marienfeld Laboratory Glassware) at 4 x 10^5^ cells/ml in six-well plates and fixed with 4% paraformaldehyde in PBS for 20 minutes at room temperature. Fixed cells were washed with PBS and permeabilized with 0.1% Triton X-100 in PBS for 5 minutes. After washing with PBS, the coverslips were incubated with BAG5 (Santa Cruz, USA)- and α-synuclein (Genetex, USA)-specific antibodies overnight at 4°C. The coverslips were incubated with Fluorescein (FITC) AffiniPure Goat Anti-Mouse IgG (H+L) (Jackson ImmunoResearch, USA) and Rhodamine Red-X-conjugated goat-anti-rabbit IgG (H+L) (Jackson ImmunoResearch, USA) overnight at 4°C. After washing twice with PBS, the coverslips were stained with DAPI for 10 minutes, and cells were mounted with mounting medium (Sigma-Aldrich, USA). Confocal images were captured under a Zeiss LSM 510 Laser Scanning Fluorescence Confocal Microscope. The percentage of cells with colocalization was determined by counting yellow blobs of at least 1,000 cells per strain using ImageJ software. Immunofluorescence images were captured under a Zeiss AxioImager M1. The percentage of cells with α-synuclein foci was determined by counting at least 1,000 cells per strain using ImageJ software.

### Coimmunoprecipitation (Co-IP)

Etoposide- or H_2_O_2_-treated cells were harvested in NP40 lysis buffer (50 mM Tris-HCL pH 7.5, 150 mM NaCl, 2 mM EDTA, 1 mM NA_3_VO_4_, 0.1 M NaF, 0.1% NP40, 1 mM PMSF). Cell lysates (1000 μg) and human brain lysates (200 μg, Novus Biological, USA) were incubated overnight with anti-BAG5 (5 μg, Santa Cruz, USA) at 4°C. Immunocomplexes were isolated with protein A-Sepharose beads saturated with 1% BSA by rotating for 5 h at 4°C. After incubating and washing the mixture, bound proteins were denatured, eluted, and resolved by 12% SDS-PAGE.

### Purification of the GST-BAG5 or GST-BAG5 (DARA) recombinant protein

The expression of the GST-BAG5 or GST-BAG5 (DARA) was performed in *Escherichia coli* BL21 (DE3) tRNA cells by induction with 0.1 mM IPTG overnight at 16°C. The cells were harvested by centrifugation and suspended in 20 mM Tris-HCl buffer (pH 8.0) containing 200 mM NaCl, 1 mg lysozyme, and 100 mM PMSF. The cells were lysed by sonication and cleared by centrifugation. The clarified lysates were isolated with GST beads by rotating for 2 h at 4°C. After incubating and washing the mixture, GST-bound BAG5 or BAG5 (DARA) proteins were eluted with 50 mM Tris-HCl buffer (pH 7.5) containing 5 mM glutathione.

### Refolding assay

U2OS, HeLa, and SH-SY5Y cells were transfected with indicated plasmids for 48 h. Cells were harvested and lysed using a Human HSP70/HSP40 Protein Refolding Kit (R&D Systems, USA) following the manufacturer’s protocol. A 4-μl aliquot of the lysate and 50 μl of Luciferin reagent were transferred to a 96-well plate with an equal amount of luciferase substrate. The experiments were performed in triplicate.

### Immunohistochemical staining

The transgenic *SNCA* p.A53T mice (B6.Cg-2310039L15RikTg(Prnp-SNCA*A53T)23Mkle/J) [36] were applied to examine the expression and localization of BAG5 in nigral dopaminergic neurons. Offspring were tail-genotyped and maintained in a specific pathogen-free facility.

Brains of transgenic *SNCA* p.A53T mice and littermate control mice were washed in PBS, fixed in 4% paraformaldehyde, cryoprotected in 30% sucrose in PBS, and embedded in OCT compound. The substantia nigra pars compacta were cut into 30-μm-thick free-floating sections (anterior-posterior (AP) -2.30 to -4.16 mm from the bregma). The sections were rinsed with PBS, incubated for 30 minutes at room temperature with a blocking solution (BSA in PBS with 0.3% of Triton X-100), and then incubated overnight at 4°C with a rabbit polyclonal antibody raised against TH (Abcam, UK), phospho-Ser129 α-synuclein (Abcam, UK) or BAG5 (Atlas Antibodies, Sweden). After washing, sections were incubated with the secondary antibody in the dark for 3 h. Quantitative analysis of BAG5, phospho-Ser129 α-synuclein, and TH-positive neurons was carried out based on the number of immunopositive cell types with clearly defined nuclei. Confocal images were captured under a LEICA SP8X Laser Scanning Fluorescence Confocal Microscope. The percentage of cells with colocalization was determined by counting yellow blobs from at least 1,000 cells per strain using ImageJ software. All experimental procedures involving animals were approved by the Institutional Animal Care and Use Committees at the Laboratory Animal Center, College of Medicine, National Taiwan University (approval number: 20190041).

### Filter-trap assay

SH-SY5Y cells were treated with 10 μM of rotenone and/or were transfected with α-synuclein mutant (A53T) plasmid or shRNA against BAG5. U2OS, HeLa, and SH-SY5Y cells were transfected with various amounts of a BAG5-expressing plasmid. Cell pellets were collected and lysed with a buffer containing 50 mM Tris, pH 7.5, 150 mM NaCl, 2 mM EDTA, 1 mM Na_3_VO_4_, and 0.1% NP40. The samples were mixed with SDS to a final concentration of 2% and filtered through a 96-well dot blot apparatus (Bio-Rad Laboratories, USA) containing a 0.2-μm nitrocellulose membrane. The nitrocellulose membrane was then probed with the anti-α-synuclein antibody (Genetex, USA). Chemiluminescence was quantified using ImageJ software.

### Statistical analysis

All experiments were performed with at least three biological repeats. Paired data are expressed as the means ± standard deviation (SD) and were analyzed using Student’s t-test with the two-tailed distribution.

## Supplementary Material

Supplementary Figures

Supplementary Tables
