# Supplementary material for: Stress-induced p53 drives BAG5 cochaperone expression to control α-synuclein aggregation in Parkinson's disease
Source: Aging (Albany NY). 2020 Oct 21;12(20):20702–27. doi: 10.18632/aging.103998 (PMC7655153; doi:10.18632/aging.103998)
Supplement: Supplementary Figures [file aging-12-103998-s001..pdf]

SUPPLEMENTARY FIGURES

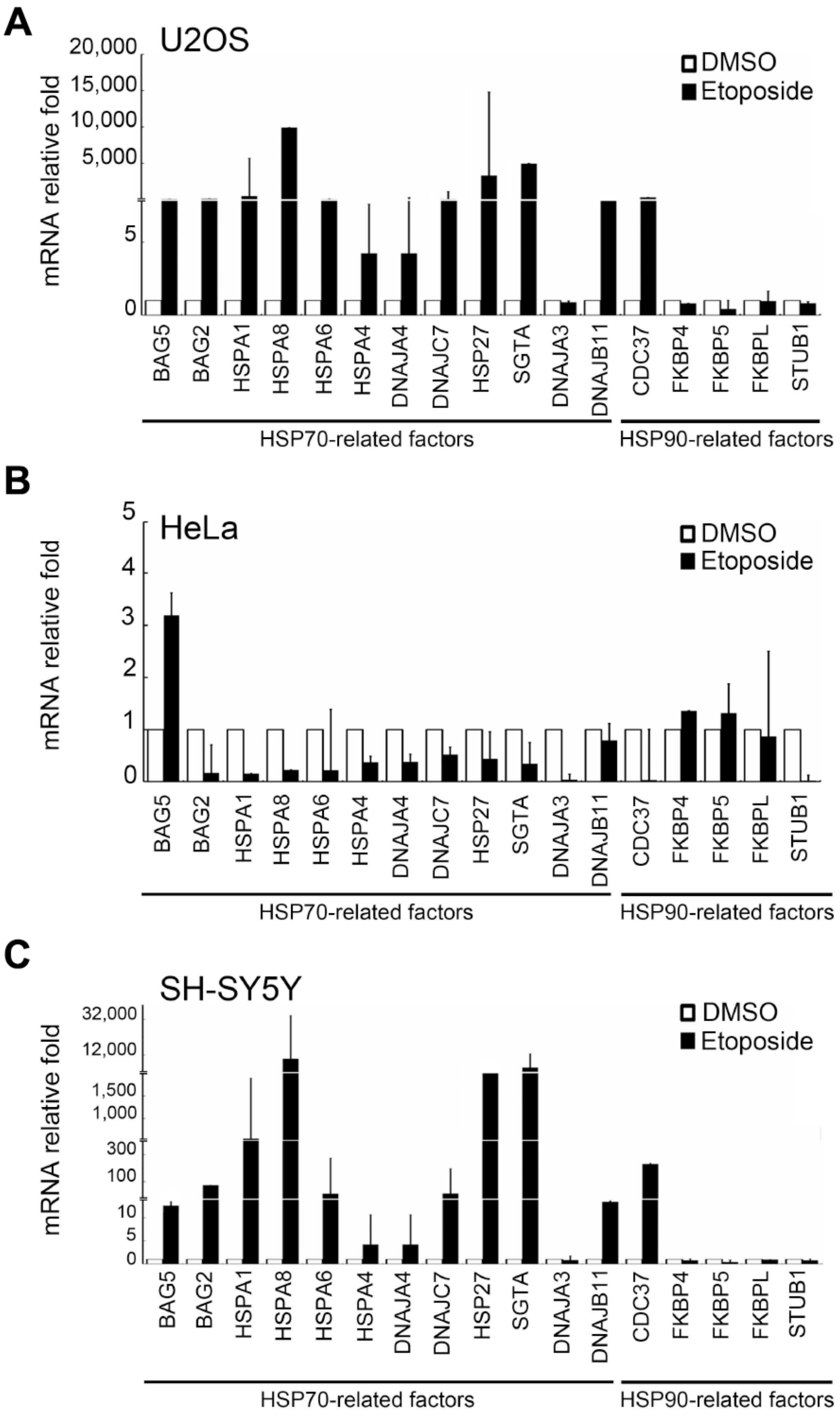

Supplementary Figure 1. BAG5 is upregulated in etoposide-treated U2OS, HeLa and SH-SY5Y cells. (**A**–**C**) U2OS, HeLa and SH-SY5Y cells were treated with 10 μM of etoposide or DMSO for 48 h. Total RNA was extracted, and transcripts of HSP70- and HSP90-related factors were detected by RT-Q-PCR. The relative fold in mRNA expression was normalized to GAPDH and DMSO. Error bars represent the standard deviation of the means calculated using data from three biologically independent experiments.

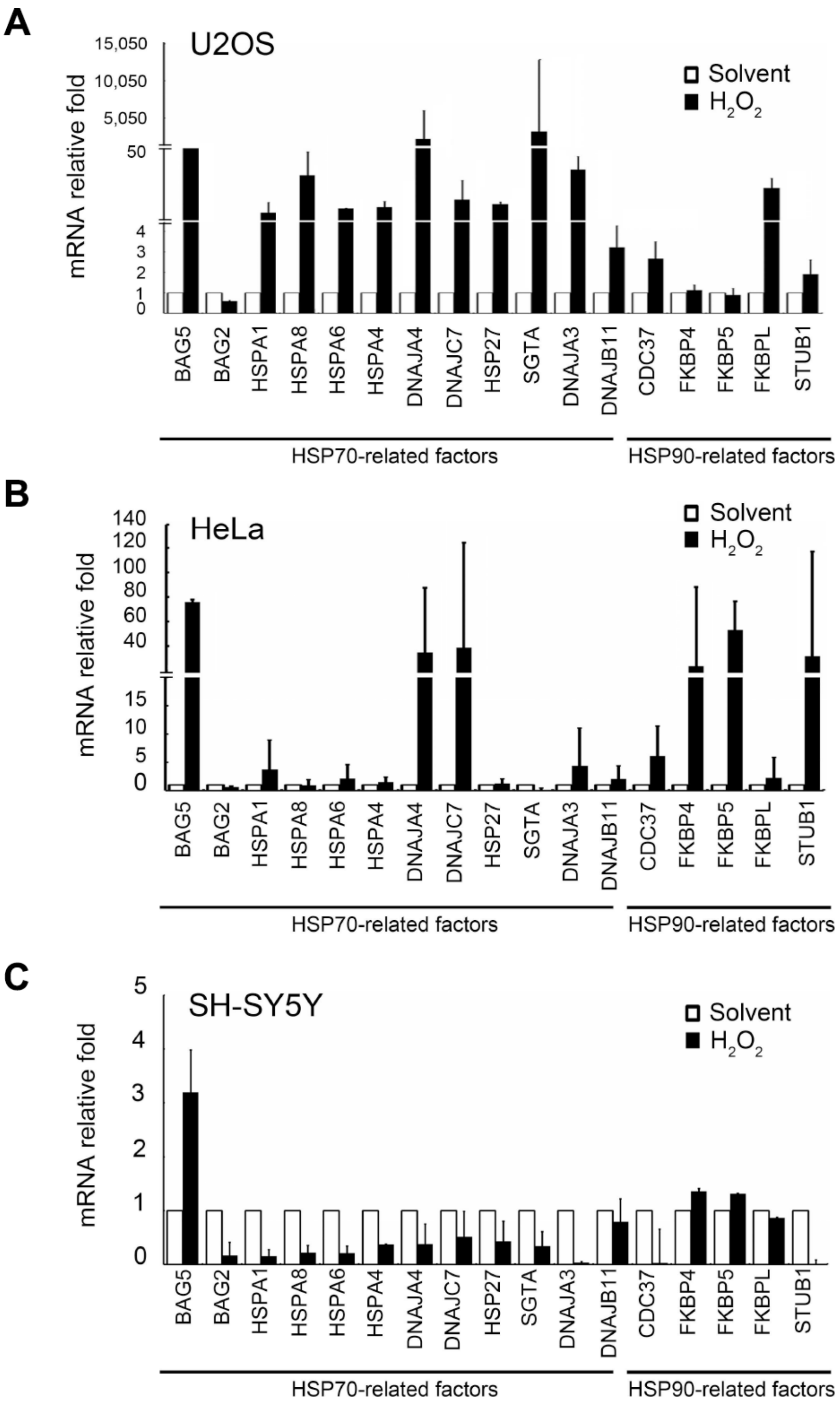

Supplementary Figure 2. BAG5 is upregulated in oxidative stress-treated U2OS, HeLa and SH-SY5Y cells. (**A**–**C**) U2OS, HeLa and SH-SY5Y cells were treated with 250 μM of H_2O_2 or solvent for 24 h. Total RNA was extracted, and transcripts of HSP70-and HSP90-related factors were detected by RT-Q-PCR. The relative fold in mRNA expression was normalized to GAPDH and the solvent control. Error bars represent the standard deviation of the means calculated using data from three biologically independent experiments.

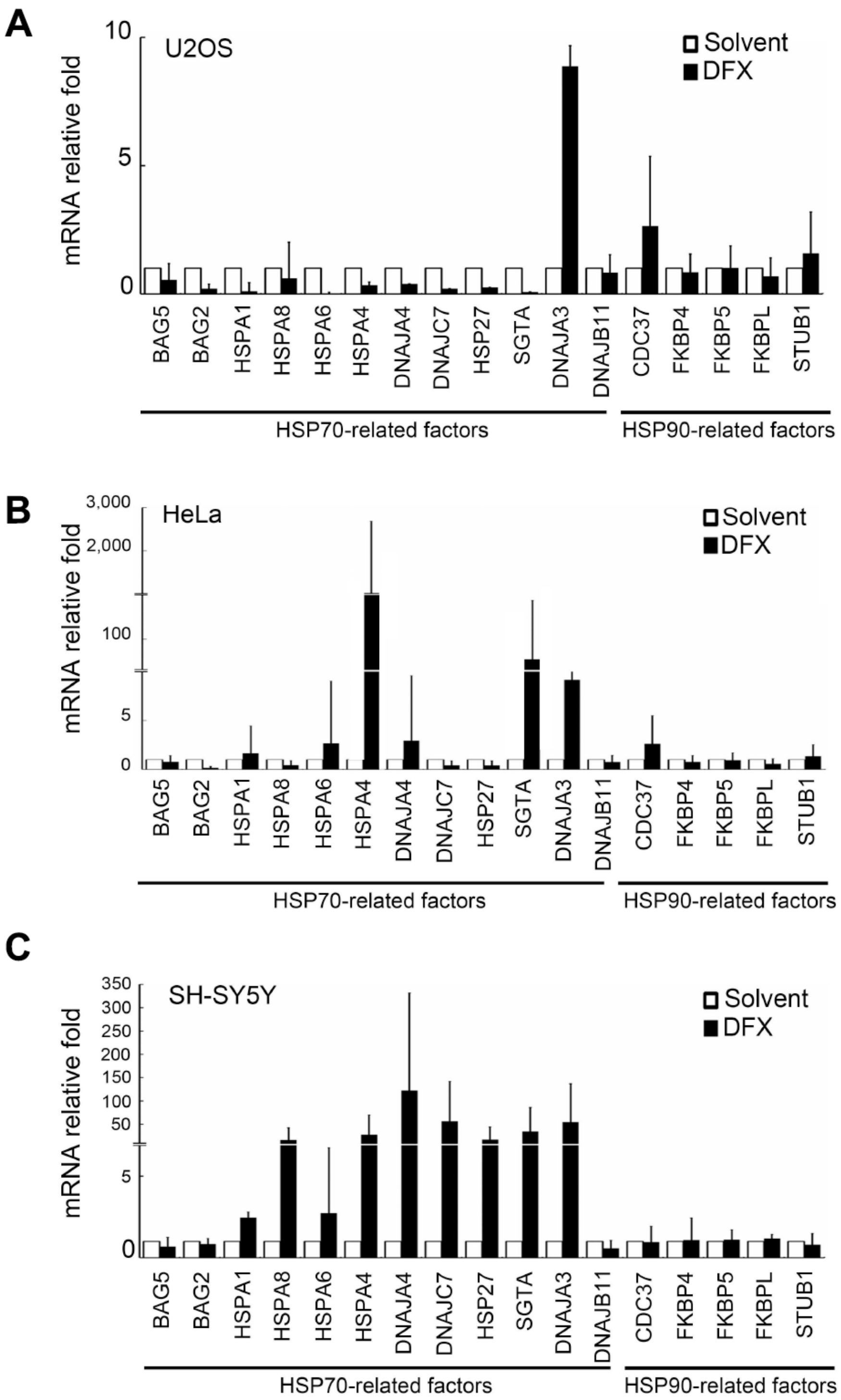

Supplementary Figure 3. Detection of cochaperones' expression in hypoxia-treated U2OS, HeLa, and SH-SY5Y cells. (**A**–**C**) U2OS, HeLa, and SH-SY5Y cells were treated 20 with 10 μM of DFX or solvent for 24 h, and total RNA was extracted. The transcripts of HSP70- and HSP90-related factors were detected by RT-Q-PCR. The relative fold in mRNA expression was normalized to those of GAPDH and the solvent control. Error bars represent the standard deviation of the means calculated using data from three biological independent experiments.

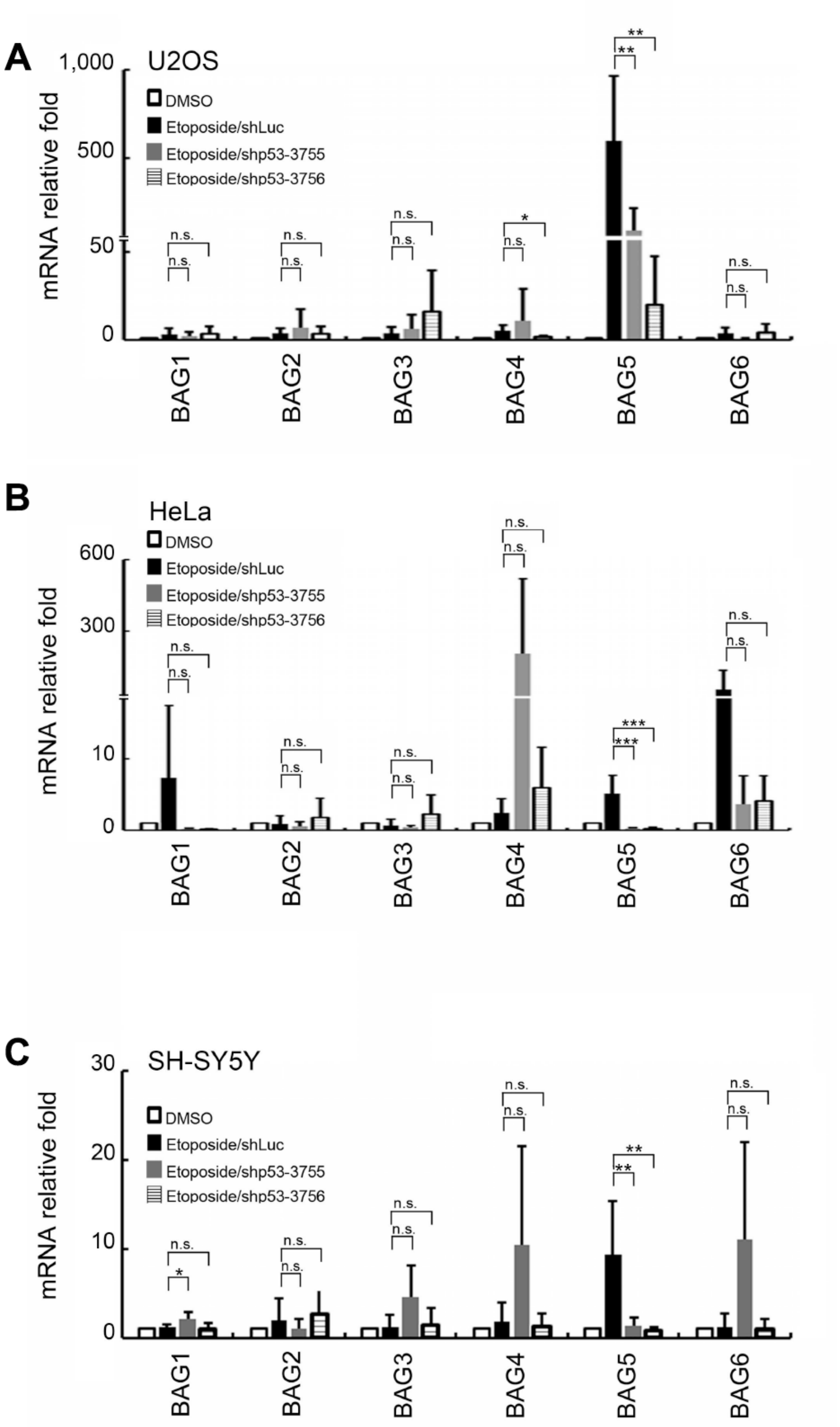

Supplementary Figure 4. Knockdown of p53 downregulates BAG5 expression in etoposide-treated cells. (**A**–**C**) U2OS, HeLa, and SH-SY5Y cells with and without p53 knockdown were treated with 10 μM of etoposide or DMSO for 48 h. Total RNA was extracted, and BAG1-BAG6 transcripts were detected by RT-Q-PCR. The relative fold in BAG mRNA expression was normalized to GAPDH. Error bars represent the SD of the means calculated using data from at least three biologically independent experiments (Student's t-test; *, $p < 0.05$, **, $p < 0.01$, ***, $p < 0.001$).

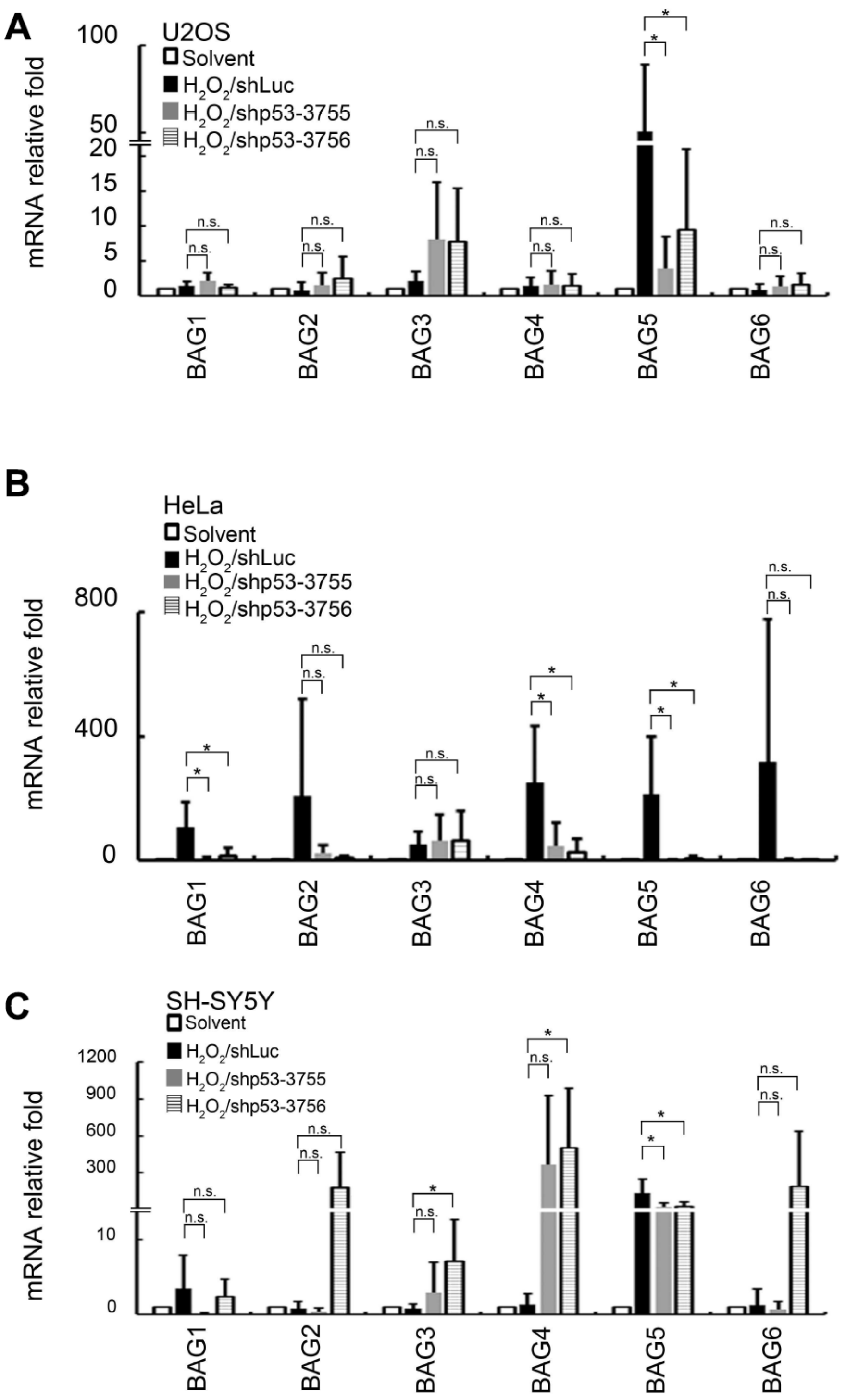

Supplementary Figure 5. The depletion of p53 reduces BAG5 expression in the oxidative stress-induced cells. (**A**–**C**) U2OS, HeLa, and SH-SY5Y cells were treated with 250 μM of H_2O_2 or solvent for 24 h, and knockdown of p53 was performed by shRNAs. Total RNA was harvested, and BAG1-BAG6 transcripts were detected by RT-Q-PCR. The relative fold in BAG mRNA transcripts was normalized to GAPDH. Error bars represent the SD of the means calculated using data from at least three biologically independent experiments (Student's t-test; *, $p < 0.05$).

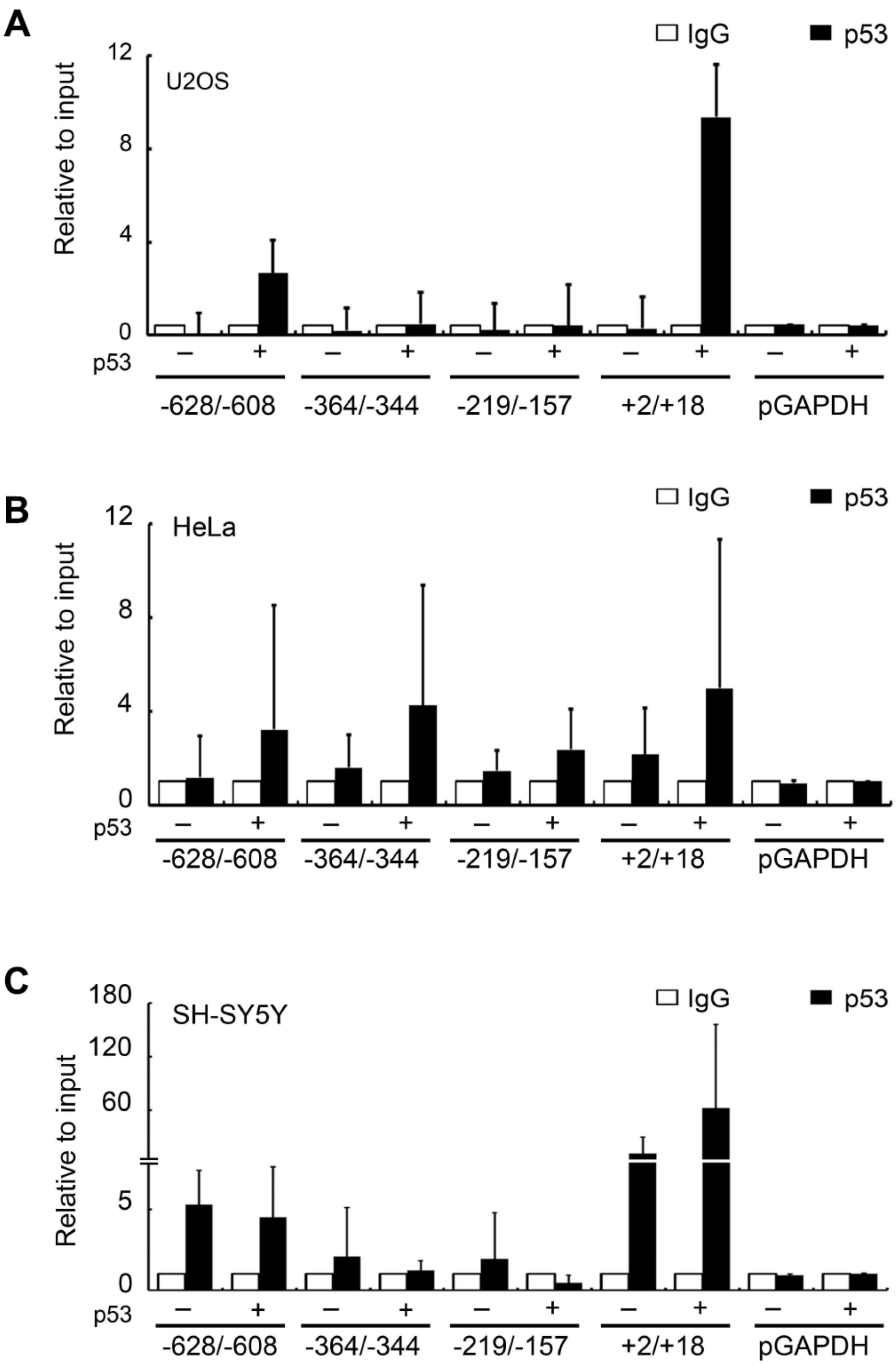

Supplementary Figure 6. p53 binds to the BAG5 promoter to facilitate the transactivation of BAG5. (**A**–**C**) U2OS, HeLa, and SH-SY5Y cells were transfected with a p53-expressing plasmid (pcDNA3.1-p53) and subjected to ChIP with p53 antibodies. Precipitated DNA was subjected to Q-PCR analysis with the primers as indicated. Each value of the bar is represented as a percentage of the respective input DNA.

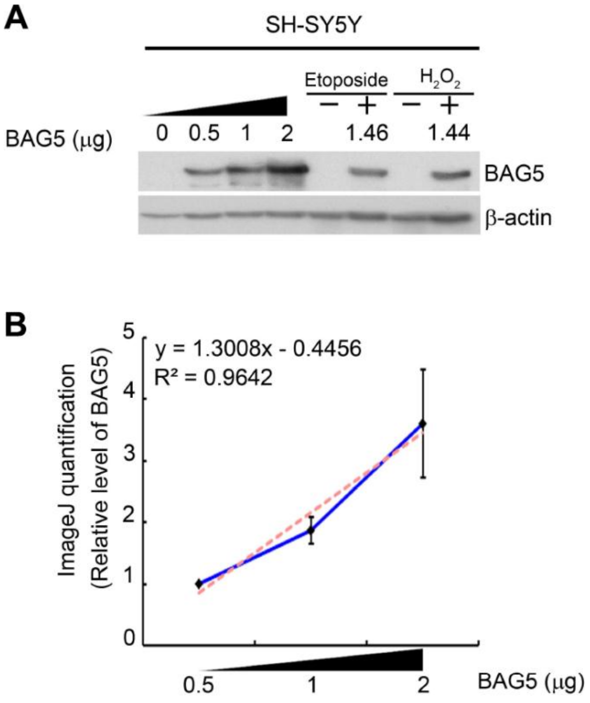

Supplementary Figure 7. The cellular amount of BAG5 in the plasmid-driven overexpression condition mimics that under stress treatment. (**A**) SH-SY5Y cells were transfected with various amounts of the pCMV-Tag2B-BAG5 plasmid or treated with 10 μM of etoposide for 48 h, 250 μM of H_2O_2 for 24 h, or solvent. Expression levels of BAG5 were detected by Western blotting. (**B**) The protein bands of Western blots were quantified by ImageJ. Linear regression was performed using scores of ImageJ for different amounts of the BAG5 expression plasmid. The amounts of etoposide- and H_2O_2-induced BAG5 were calculated by the linear regression equation. The red dashed line indicates the trendline, and the blue solid line indicates the relative level of BAG5 signal normalized by β-actin. Error bars represent the SD of the means calculated using data from three independent experiments.

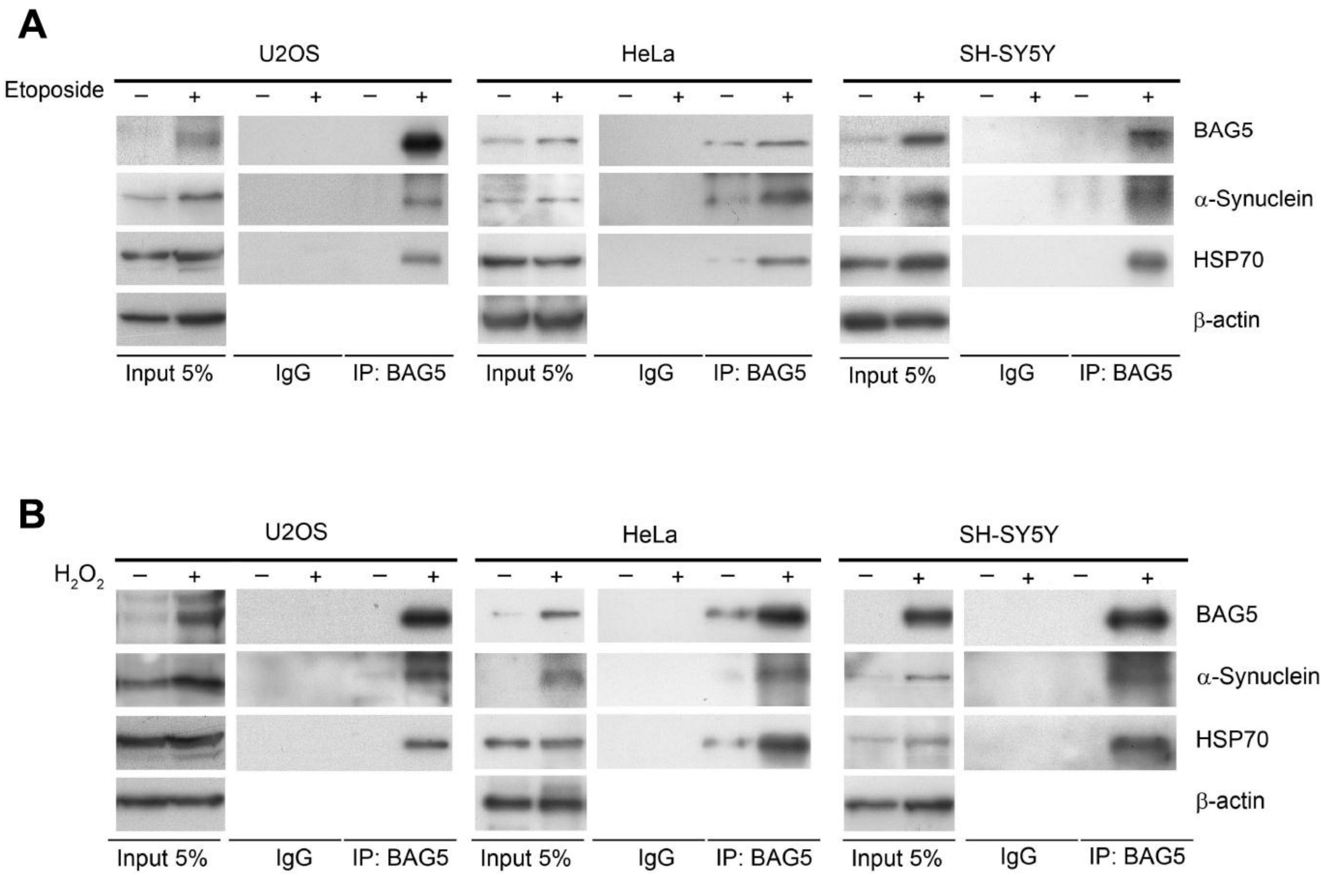

Supplementary Figure 8. BAG5 interacts with α-synuclein and HSP70 in stress stimulated cells. U2OS, HeLa, and SH-SY5Y cells were treated with 10 μM of etoposide for 48 h (**A**), 250 μM of H_2O_2 for 24 h (**B**), or solvent. Immunoprecipitations were performed with an anti-BAG5 antibody. Immunoprecipitates were sequentially probed with anti-α-synuclein (upper) and anti-HSP70 (middle) antibodies. Five percent of lysates used for immunoprecipitation was loaded as the input and probed with anti-BAG5 (upper), anti-α-synuclein (middle), and anti-HSP70 (lower) antibodies. β-Actin was used as a loading control.
