# Supplementary material for: Stress-induced p53 drives BAG5 cochaperone expression to control α-synuclein aggregation in Parkinson's disease
Source: Aging (Albany NY). 2020 Oct 21;12(20):20702–27. doi: 10.18632/aging.103998 (PMC7655153; doi:10.18632/aging.103998)
Supplement: Supplementary Tables [file aging-12-103998-s002..pdf]

SUPPLEMENTARY TABLE

Supplementary Table 1. Oligo sequences used for gene knockdown and primers used in this study.

Oligo sequences used for gene knockdown			
**Clone ID**	**Gene Symbol**	**Sequence**	**Region**
TRCN0000003755	*p53*	GTCCAGATGAAGCTCCCAGAA	CDS
TRCN0000003756	*p53*	CACCATCCACTACAACTACAT	CDS
TRCN0000033583	*BAG5*	GCAGGGAGAAGAGAAGTGTAA	CDS
TRCN0000299795	*BAG5*	GCAGACACAACTAAAGCATTT	CDS

Primers used in this study			
**Genes**	**Forward sequence**	**Reverse sequence**	**Assay**
BAG5	ACCCACACCGGATTGAAATA	TTATCTCTTGCACCGCACAG	Bio-Rad qPCR
GAPDH	GAAGGTGAAGGTCGGAGTC	GAAGATGGTGATGGGATTTC	Bio-Rad qPCR
BAG2	AGGCGAAGATCAACGCTAAA	CTTGGCATTTCCCAAATCAT	Bio-Rad qPCR
CDC37	TCCAGAAGTGCTTCGATGTG	TCTTGGGAACAGCTTCCAGT	Bio-Rad qPCR
FKBP4	GCGGTGAAGGCTATGCTAAG	CATTTGGTGGGATTTGGAAC	Bio-Rad qPCR
FKBP5	TCCCTCGAATGCAACTCTCT	GCCACATCTCTGCAGTCAAA	Bio-Rad qPCR
FKBPL	CTCCTACCGAAACGCTTGAG	GCCTAGTTTGGGTTTGTCCA	Bio-Rad qPCR
STUB1	CGACTACCTGTGTGGCAAGA	CAAGTTGGGGATGAGCTGTT	Bio-Rad qPCR
DNAJA3	AACAGCTACGGCTACGGAGA	GAAAGGAATCCCTCCTCGTC	Bio-Rad qPCR
DNAJB11	TGGGGATTTTGGTTTCATGT	AGCGTTCGTTCTTCATTCA	Bio-Rad qPCR
HSPA1	CGACCTGAACAAGAGCATCA	AAGATCTGCGTCTGCTTGGT	Bio-Rad qPCR
HSPA8	GGAGGTGGCACTTTTGATGT	AGCAGTACGGAGGCGTCTTA	Bio-Rad qPCR
HSPA6	GGCAAGGAGCTGAACAAGAG	GAAAGTCTGGGTCTGCTTGG	Bio-Rad qPCR
HSPA4	AGCCAAGAAGGCAAAAGTGA	CCACTGCGTTCTTAGCATCA	Bio-Rad qPCR
DNAJA4	CTACAAAGCACCCCTGGAAA	AGCTCCACCTGATCCATGTC	Bio-Rad qPCR
DNAJC7	AGCAATGCTGGGTCGTTATC	AGCCATCCTGAGAGCCTGTA	Bio-Rad qPCR
HSP27	GGACGAGCATGGCTACATCT	GACTGGGATGGTGATCTCGT	Bio-Rad qPCR
SGTA	TTGGGGTGACGGTAGAAGAC	GTTGAGCTCGATGGCTTTTC	Bio-Rad qPCR
BAG1	GGGGTTCCACAGTCTTTTCA	GAGCTTCAGCTTGCAAATCC	Bio-Rad qPCR
BAG3	GGAGTGCTGAAAGTGGAAGC	CTGGACTTGACCTGGGACAT	Bio-Rad qPCR
BAG4	CAGCCCAACTCCAGTCTCTC	CTGATGAAGGCCAGGGATAA	Bio-Rad qPCR
BAG6	TGAGCTGGCTGACCACTATG	GCCTCTTCTGCTGTTGTTCC	Bio-Rad qPCR
ChIP -628/-608	GGCTGTGAGTCTGAGGTGTG	CAGCTACTCGGGAGGTTGAG	ChIP -736/-720 bp
ChIP -364/-344	TGTTGGGACACAGGTGTGAG	CACCCCCATCACACACATAC	ChIP -441/-382 bp
ChIP -219/-157	TGTAAGGAGGTGAGGGTGGT	TATTTGAGACCCAGGCATCC	ChIP -323/-236 bp
ChIP+2/+8	CCGTCTTCACTGCTTTGTGA	AGACAAACAACTGCCCGAAG	ChIP -57/+18 bp
p*GAPDH*	AGCTCAGGCCTCAAGACCTT	AAGAAGATGCGGCTGACTGT	ChIP pGAPDH
